# Crystal Structure of the *Streptomyces coelicolor* Sortase E1 Transpeptidase Provides Insight into the Binding Mode of the Novel Class E Sorting Signal

**DOI:** 10.1371/journal.pone.0167763

**Published:** 2016-12-09

**Authors:** Michele D. Kattke, Albert H. Chan, Andrew Duong, Danielle L. Sexton, Michael R. Sawaya, Duilio Cascio, Marie A. Elliot, Robert T. Clubb

**Affiliations:** 1 Molecular Biology Institute, University of California, Los Angeles, Los Angeles, California, United States of America; 2 Molecular Biology Interdepartmental Program, University of California, Los Angeles, Los Angeles, California, United States of America; 3 Department of Pharmacology, Yale University School of Medicine, New Haven, Connecticut, United States of America; 4 Department of Biology and Michael G. DeGroote Institute for Infectious Disease Research, McMaster University, Hamilton, Ontario, Canada; 5 Department of Chemistry and Biochemistry, University of California, Los Angeles, Los Angeles, California, United States of America; 6 UCLA-DOE Institute of Genomics and Proteomics, University of California, Los Angeles, Los Angeles, California, United States of America; University of Texas Medical School at Houston, UNITED STATES

## Abstract

Many species of Gram-positive bacteria use sortase transpeptidases to covalently affix proteins to their cell wall or to assemble pili. Sortase-displayed proteins perform critical and diverse functions for cell survival, including cell adhesion, nutrient acquisition, and morphological development, among others. Based on their amino acid sequences, there are at least six types of sortases (class A to F enzymes); however, class E enzymes have not been extensively studied. Class E sortases are used by soil and freshwater-dwelling Actinobacteria to display proteins that contain a non-canonical LAXTG sorting signal, which differs from 90% of known sorting signals by substitution of alanine for proline. Here we report the first crystal structure of a class E sortase, the 1.93 Å resolution structure of the SrtE1 enzyme from *Streptomyces coelicolor*. The active site is bound to a tripeptide, providing insight into the mechanism of substrate binding. SrtE1 possesses β3/β4 and β6/β7 active site loops that contact the LAXTG substrate and are structurally distinct from other classes. We propose that SrtE1 and other class E sortases employ a conserved tyrosine residue within their β3/β4 loop to recognize the amide nitrogen of alanine at position P3 of the sorting signal through a hydrogen bond, as seen here. Incapability of hydrogen-bonding with canonical proline-containing sorting signals likely contributes to class E substrate specificity. Furthermore, we demonstrate that surface anchoring of proteins involved in aerial hyphae formation requires an N-terminal segment in SrtE1 that is presumably positioned within the cytoplasm. Combined, our results reveal unique features within class E enzymes that enable them to recognize distinct sorting signals, and could facilitate the development of substrate-based inhibitors of this important enzyme family.

## Introduction

Gram-positive bacteria productively interact with their environment via surface displayed proteins anchored by sortase enzymes [1–10]. These cysteine transpeptidases modulate the functionality of bacterial surfaces by affixing proteins that perform a variety of functions, including cell adhesion, nutrient acquisition, immune evasion, aerial hyphae development and sporulation, among others [3,4,9,11]. Understanding the mechanism of catalysis and substrate recognition is of prime interest, as small molecule sortase inhibitors could have potent, anti-infective properties against pathogenic microbes by preventing them from displaying virulence factors [12–17]. Moreover, sortase-mediated protein ligation is an emerging biotechnology tool to modify and immobilize proteins, and a greater understanding of how these enzymes recognize their substrates could facilitate their rational engineering [18–27].

The catalytic mechanism of the *Staphylococcus aureus* sortase A enzyme (SaSrtA) has been characterized in detail and is paradigmatic [2]. SaSrtA, a class A enzyme, covalently anchors proteins to the cell wall by catalyzing a transpeptidation reaction that joins its protein substrate to the crossbridge peptide present in lipid II [28–30]. An N-terminal transmembrane (TM) segment positions SaSrtA at the cell membrane where it recognizes protein substrates via their C-terminal, cell wall sorting signal (CWSS). The CWSS consists of a LPXTG pentapeptide sorting signal motif (where X is any amino acid), followed by a hydrophobic segment that is embedded in the bilayer and a C-terminal cluster of positively-charged amino acids [28]. A conserved catalytic triad (His120, Cys184, Arg197) is required for transpeptidation activity in SaSrtA; this reaction is catalyzed through a ping-pong mechanism in which its active site cysteine residue nucleophilically attacks the backbone carbonyl carbon of the threonine residue within the LPXTG motif. Cleavage of the scissile T-G peptide bond forms a long-lived, sortase-protein thioacyl intermediate [31,32]. The thioacyl bond is then nucleophilically attacked by the amino group located in lipid II, creating a peptide bond-linked, protein-lipid II product [29,30,33]. The transpeptidation product is subsequently incorporated into the peptidoglycan via the conventional transglycosylation and transpeptidation reactions that synthesize the cell wall. All sortases are believed to catalyze transpeptidation reactions through a similar mechanism.

At present, over 1,800 gene sequences encoding sortase enzymes have been identified within ~600 species of bacteria [34]. Members of the sortase superfamily are predominantly found in Gram-positive bacteria and are grouped into distinct classes based on their amino acid sequences (class A to F enzymes) [3,35,36]. Biochemical and bioinformatics analyses suggest that class A, B, C, D and E enzymes have evolved specificities for LPXTG, NPXTN, LPXTG, LPXTA and LAXTG sorting signals, respectively (differences from LPXTG underlined). Most microbes express more than one type of sortase, which function non-redundantly to “sort” distinct proteins to the cell surface by recognizing their class-specific sorting signals. At present, atomic structures of class A, B, C, and D enzymes have been reported, revealing class-specific structural features [6]. Several studies using substrate analogues have also revealed how class A and B enzymes recognize their sorting signals [37–40]. However, the structure of a class E enzyme, or the mechanism though which it recognizes the unique LAXTG sorting signal substrate is not known.

Class E sortases are prevalent in soil-dwelling and aquatic actinobacteria (e.g. *Corynebacterium* and *Streptomyces* genera) [36]. *Streptomyces coelicolor* is one of the best-studied members of the Actinobacteria and uses two Class E enzymes to decorate its surface [11,41]. It exhibits a complex life cycle that has three morphologically distinct stages: vegetative hyphae, aerial hyphae, and spores. *S*. *coelicolor* is predicted to encode an astounding seven sortase enzymes: two class E and five class F enzymes. The class E enzymes in *S*. *coelicolor* (called SrtE1 and SrtE2) anchor chaplin proteins to the cell surface (ChpA, ChpB and ChpC) that function to promote the transition from vegetative growth to aerial hyphae formation. Strong evidence supports the notion that they recognize an unusual LAXTG sorting signal, as Duong et al. showed that SrtE1 and SrtE2 selectively cleave LAXTG-containing peptides *in vitro* and that they attach the ChpC protein bearing this motif to the cell wall [11,42]. These recognition events play a critical role in the lifecycle of this microbe as a *srtE1srtE2-* double mutant is delayed in aerial hyphae formation, is unable to sporulate, and fails to display chaplins on its aerial surfaces. Here we report the first atomic structure of a class E sortase, SrtE1 from *S*. *coelicolor*. The crystal structure, combined with biochemical, computational and cellular studies, provides insight in the mechanism of LAXTG sorting signal recognition.

## Results and Discussion

Actinobacteria display proteins on their cell surface using class E sortase enzymes that are distinct from other classes of sortase and have not been structurally characterized. To gain insight into their function, we investigated the class E enzymes from *S*. *coelicolor*, SrtE1 and SrtE2. We explored the importance of a unique cytoplasmic region in SrtE1 function and conducted structure-function studies of its catalytic domain to learn how it recognizes the novel LAXTG sorting signal.

### Regions within the cytoplasmic tail of SrtE1 are important for its function in vivo

**Fig 1:**
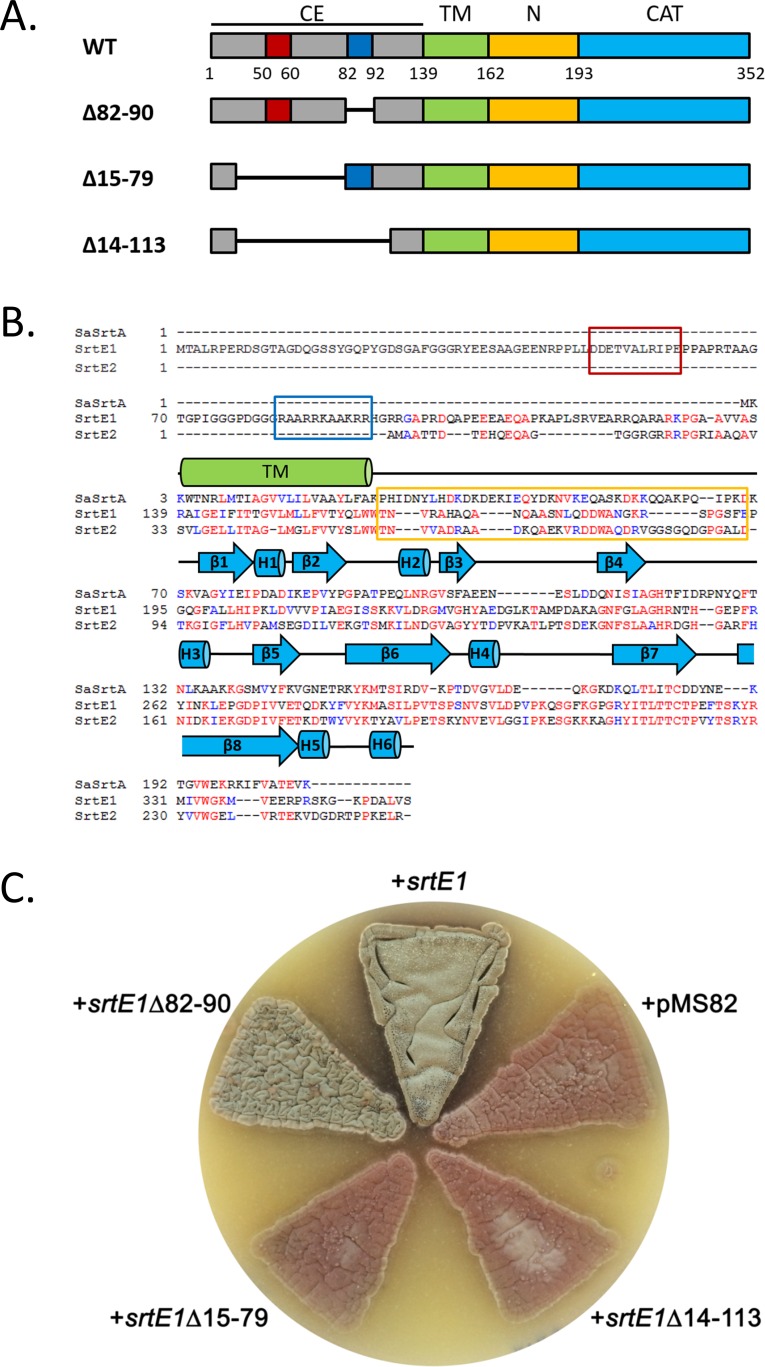
Phenotypic effect of deletions within the N-terminal cytoplasmic tail of SrtE1. A) SrtE1 constructs indicating deletion within the conserved, N-terminal cytoplasmic tail. The residue number that initiates or terminates deleted segments or domain boundaries are shown below the WT construct. The conserved acidic region (*red*) and basic region (*blue*) of the SrtE1 N-terminal cytoplasmic tail are indicated within the CE. WT, wild type; CE, cytoplasmic extension; TM, transmembrane helix; N, N-terminal membrane linker segment; CAT, catalytic core domain. B) Multiple sequence alignment of SrtE1 and SrtE2 from *S*. *coelicolor* with *S*. *aureus* SrtA. Sequence alignment was generated using the ClustalOmega server [43]. The bacterial species and accession numbers of the amino acid sequences used for the alignment are as follows: *Streptomyces coelicolor* (NP_628038 and NP_628037) and *Staphylococcus aureus* (WP_000759361). Conserved residues are indicated in *red*, and related amino acids are indicated in *blue*. Secondary structure elements from the SrtE1 crystal structure are shown in *blue* for β-strands (*arrows*) and helices (*cylinders*). The transmembrane (TM) region predicted by the THMHH server is indicated with a *green cylinder*. The conserved acidic region (*red box*) and basic region (*blue box*) within the N-terminal cytoplasmic tail of the SrtE1 enzyme, as well as conserved N-terminal membrane linker segment of SrtE1 and SrtE2 (*orange box*) are indicated. C) Effect of SrtE1 mutation on aerial development and sporulation in *S*. *coelicolor*. *srtE1* variants lacking the conserved acidic region (∆15–79), basic region (∆82–90), or both (∆14–113) were introduced into a strain of *S*. *coelicolor* lacking both *srtE1* and *srtE2* (+pMS82). Wild type *srtE1* alone (+*srtE1*) can restore aerial development and sporulation.

SrtE1 and SrtE2 contain a conserved C-terminal sortase catalytic domain (CAT) that is connected by ~30 non-polar amino acids (N) to a putative transmembrane (TM) helix (Fig 1A and 1B). The sequence of SrtE1 also contains an N-terminal extension that presumably resides within the bacterium’s cytoplasm (CE in Fig 1A). SrtE1 homologues of other actinobacteria in the streptomycetes genus also contain the cytoplasmic extension, which varies in length from ~100–200 amino acids (*e*.*g*. *Actinobacteria bacterium* and *Actinospica acidophila*) (S1 Fig). Notably, the CE segment is absent in nearly all other types of sortase enzymes, including the prototypical SaSrtA enzyme. While the sequence of the N-terminal CE segment in SrtE1 and its homologues varies considerably, two regions contain amino acids with similar physiochemical properties (S1 Fig). In SrtE1, the first conserved region corresponds to a short segment that is enriched for acidic amino acids (D50-E60) (enclosed in red box in S1 Fig), while a second region comprises a ~10 residue segment that is enriched with basic amino acids (R82-R92) (enclosed in blue box in S1 Fig). In addition, the ~25 residues that immediately precede the TM helix are also well conserved.

To probe the importance of the SrtE1 N-terminal cytoplasmic extension, we employed a mutagenic strategy to investigate its function. We first removed the nucleotides specifying residues G14-K113 of the *S*. *coelicolor* SrtE1-encoding gene (∆14–113), which encompassed both the conserved acidic and basic patches (Fig 1A). This *srtE1* deletion variant was introduced into a strain lacking both *srtE1* and *srtE2* to test its ability to complement the sortase mutant phenotype (wild type *srtE1* alone can restore aerial development and sporulation). Interestingly, loss of this region results in a failure to promote sporulation in the sortase mutant, suggesting that it contains segments important for SrtE1 function (Fig 1C). As the basic patch is the most conserved sequence within this region, we set out to determine whether it is a critical functional determinant. We deleted residues R82-R90 (including five positively charged residues) and introduced the resulting construct into our sortase mutant (∆82–90) (Fig 1A). This construct effectively restores aerial development and sporulation, suggesting this positively charged region is not important for function (Fig 1C). Finally, we created a third deletion variant lacking residues D15-G79, which included the acidic, negatively charged region (∆15–79) (Fig 1A). This SrtE1 variant is unable to function in place of wild type SrtE1 when introduced into the sortase mutant strain. Instead, it produces a phenotype that is indistinguishable from that of a complete null mutant, suggesting that segments within the first ~80 amino acids are important for SrtE1 function (Fig 1C). While we cannot rule out the possibility that this region merely contributes to the stability of SrtE1 (we were unable to generate a functional, tagged SrtE1 fusion protein *in vivo* to follow by immunoblotting), we speculate that this cytoplasmic tail, and particularly the negatively charged region, may promote interaction with other proteins in the membrane or cytoplasm.

### The crystal structure of SrtE1 reveals unique Class E features

**Fig 2:**
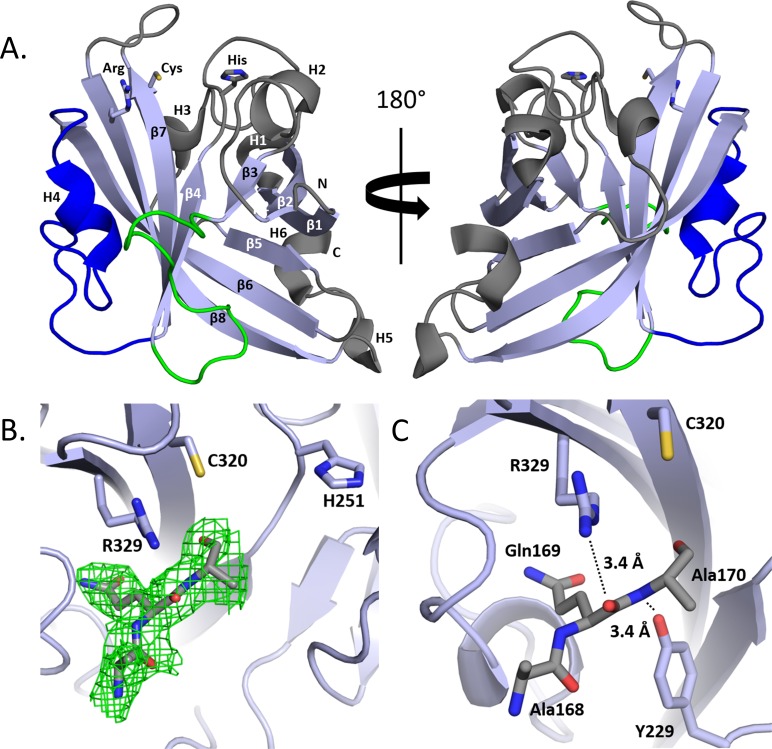
Crystal structure of SrtE1 from *S*. *coelicolor*. A) Ribbon diagram of the SrtE1catalytic domain with the conserved Arg-Cys-His triad shown in sticks. Beta sheets (β) and helices (H), as well as N- and C-termini, are labeled accordingly. The sortase β-barrel core (*light blue*), structurally unique β3/β4 (*green*) and β6/β7 loops (*blue*), and accessory loops and helices (*gray*) are colored. B) An F_o_−F_c_ omit map of the active site contoured at +3 σ (*green mesh*). The map was generated by omitting the AQA tripeptide from the final model and performing additional refinement. The omit density accommodates an AQA tripeptide adjacent to Arg329 and Cys320 within the active site. C) Interactions between SrtE1 active site residues and AQA tripeptide. Potential hydrogen bond interactions (*black dashed line*) between R329, Y229, and AQA tripeptide (*sticks*) are shown.

We determined the 1.93Å resolution crystal structure of the C-terminal region of SrtE1 (SrtE1^ΔN^, residues T162-S352), which contains the conserved extracellular catalytic domain and a ~30 amino acid N-terminal linker segment that connects this domain to the putative TM anchor. Crystallization of SrtE1^ΔN^ was challenging as the protein irreversibly precipitated at low concentrations (~7 mg/ml) within a few days of storage at 4°C. However, for one condition in our screen, diffracting crystals formed after ~3–4 weeks from a dense precipitate and proteinaceous skin within the hanging drop. Attempts to reproduce crystal growth for structural characterization of enzyme-substrate complexes proved unsuccessful. SrtE1^ΔN^ crystallized in the C222_1_ space group, with a single protein molecule in the asymmetric unit. The structure was determined by molecular replacement, and is well-defined by continuous electron density for residues forming the catalytic domain (P193-V351) (Fig 2). However, no density was observed for the ~30 amino acids in the N-terminal linker segment that precedes the catalytic domain (T162-E192), except for residues A168-A170 (described in detail below). Complete data collection and structural statistics are provided in Table 1.

**Table 1:** Data collection and structure refinement statistics.

	SrtE1
**Data collection**	
Space group	C222_1_
Cell dimensions	
*a*, *b*, *c* (Å)	53.11, 104.30, 79.02
α, β, γ (°)	90.0, 90.0, 90.0
Resolution (Å)	43.53–1.93 (1.98–1.93)^b^
*R*_merge_ (%)^a^	19.0 (83.8)
CC1/2 (%)	98.7 (77.1)
*I* / σ*I*	6.82 (2.0)
Completeness (%)	97.5 (99.9)
Redundancy	5.9 (4.7)
Wilson B-factor (Å^2^)	26.2
**Refinement**	
Resolution (Å)	43.5–1.93
No. reflections	17583
*R*_work_ / *R*_free_ (%)^c^	19.9/22.9
No. atoms	1283
Protein	1232
Ligand/ion	14
Water	37
*B*-factors (all atoms)	33.7
Protein	32.9
Ligand/ion	98.6
Water	33.6
R.m.s. deviations	
Bond lengths (Å)	0.007
Bond angles (°)	1.089
Ramachandran favored (%)	98.0
Ramachandran allowed (%)	2.0
Ramachandran generally allowed (%)	0.0
Ramachandran outliers (%)	0.0

^a^ Data from two crystals were merged for structure determination.

^b^ Values in parentheses are for highest-resolution shell.

^c^ R_free_ calculated using 5% of the data.

SrtE1^ΔN^ is monomeric and adopts a classical sortase-like *β-*barrel fold, but contains unusual β3/β4 and β6/β7 loops, as well as several accessory helices (Fig 2A). The catalytic domain starts with strand β1 (residues G196-I202), followed by a short helix H1 (residues P203-D206). The chain then forms a turn such that residues in the following strand β2 (residues V207-E213) interact with the β1 strand in an antiparallel manner. A 13-amino acid loop with an ordered helix H2 (residues V220-G224) then leads into strand β3 (residues V226-H228), which lays parallel to strand β2. A 16-amino acid loop precedes strand β4 (residues G244-A249), which interacts in an antiparallel manner with strand β3. The conserved active site histidine residue, His251, immediately follows strand β4 and is located on a long segment intervening between β4 and β5 that contained helix H3 (residues Y261-L265). Strand β5 (residues P270-E274) then aligns with strand β1 in an antiparallel manner, followed by a short turn that directs strand β6 (residues K278-T291) towards the active site. An extended, 26-residue loop containing an ordered 3_10_ helix H4 (residues N295-D300) leads into strand β7 (residues R313-T321), which interacts in a parallel manner with strand β4. The conserved catalytic cysteine residue (C320) is positioned at the end of the β7 strand, followed by a loop that reverses the direction of the polypeptide chain to lead into strand β8 (residues Y328-P341). The β8 strand contains a conserved active site arginine residue (R329) and is positioned antiparallel with respect to strands β6 and β7. Two short helices, H5 (residues S343-G345) and H6 (residues P347-V351), complete the structure.

During protein structure refinement, positive difference density within the active site clearly outlined the shape of a tripeptide. The sequence Ala-Gln-Ala (AQA) fit the density well (Fig 2B). This sequence assignment is consistent with residues Ala168-Ala170 in the N-terminal linker of SrtE1^ΔN^ that precede the catalytic domain. Ala168 in the tripeptide is positioned farthest from the active site near the β3/β4 and β6/β7 loops (Fig 2C). The remainder of the tripeptide projects towards the active site with the side chain of Gln169 contacting hydrophobic residues in the β6/β7 loop (P293 and V298) and β7 strand (I331), and the side chain of the C-terminal Ala170 pointing towards the β2/H2 loop where it engages in hydrophobic interactions with Ala249 located in strand β4. Two potential enzyme-peptide hydrogen bonds are possible: the active site Arg329 side chain may form a hydrogen bond to the backbone carbonyl oxygen of Glu169 (N-O distance 3.4 Å), and the side chain hydroxyl oxygen of Tyr229 located in the β3/β4 loop may form a hydrogen bond to the backbone amine of Ala170 (O-N distance 3.4 Å). Unlike the AQA tripeptide, electron density for residues Gln171-Glu192 that separated Ala168-Ala170 from the structured catalytic domain was not observed. The notion that these residues in the N-terminal linker are structurally disordered or undergo exchange between two or more distinct conformational states, consistent with a “lid” function observed for class C enzymes, is supported by a two dimensional (2D) ^1^H-^15^N heteronuclear single quantum coherence (HSQC) spectrum of SrtE1^ΔN^, which lacks cross peaks for ~30 residues (S2 Fig). However, more conclusive NMR evidence could not be obtained as the protein construct was unstable, making it difficult to obtain sequence specific resonance assignments. The elevated B-factors of the tripeptide suggest that the peptide may not be bound at full occupancy throughout the crystal, possibly due to incomplete proteolytic cleavage to produce the tripeptide, or constraints of crystal packing. However, the contacts observed appear chemically reasonable, suggesting a degree of biological relevance. Interestingly, the amino acid sequence of the tripeptide is present in many SrtE1 enzymes (S1 Fig), but not conserved in SrtE2 or other class E enzymes (Fig 1B).

**Fig 3:**
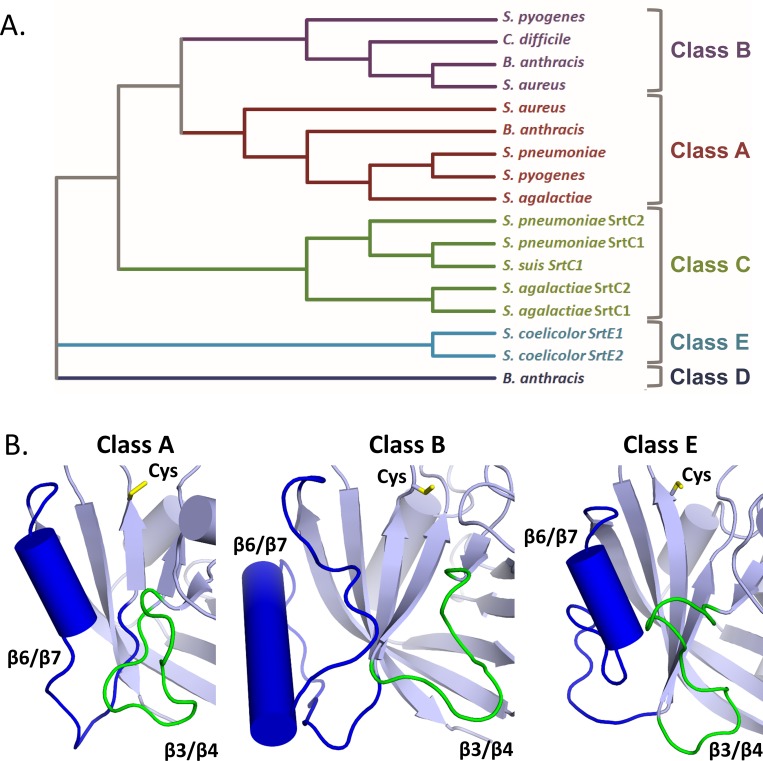
The structure of SrtE1 reveals unique features within Class E sortases. A) Phylogenetic tree of distinct sortase classes. The full amino acid sequences of 17 structurally characterized sortase enzymes were aligned with the sequences of SrtE1 and SrtE2 using the MUSCLE server and submitted to the ClustalOmega program for phylogenetic tree generation via the neighbor joining method [43,45]. The bacterial species and accession numbers of the amino acid sequences used are as follows: *Streptomyces coelicolor* (NP_628038; NP_628037), *Bacillus anthracis* (WP_011732503; WP_000093563; WP_000771607), *Streptococcus pyogenes* (WP_002984641; WP_010921812), *Clostridioides difficile* (WP_021376017), *Staphylococcus aureus* (WP_000759361; WP_054104750), *Streptococcus agalactiae* (WP_017646311; WP_000529911; WP_000746226), *Streptococcus pneumoniae* (WP_000078846; WP_001140539; ABS82110), and *Streptococcus suis* (WP_012027975). B) Sortase class comparison of distinguishing class E structural features. Class A and class B enzymes from *S*. *aureus* and a class E enzyme (SrtE1) from *S*. *coelicolor* are shown in *cartoon* with helices (*cylinders*), β3/β4 loops (*green*), β6/β7 loops (*blue*), and active site cysteine residue (*yellow*) indicated. Inspection of the most structurally related class C (PDB ID: 3RE9) and class D (PDB ID: 2LN7) enzymes, as determined by DALI analysis, revealed β3/β4 and β6/β7 loops that align similarly to the class A enzyme.

SrtE1 contains two active site loops that are structurally distinct from class A, B, C, and D sortases. Structures of representative class A, B, C and D enzymes have been determined previously [6]. As shown in Fig 3A, a phylogenetic comparison to these enzymes reveals that the sequences of the class E sortases from *S*. *coelicolor*, SrtE1 and SrtE2, differ substantially. Furthermore, a detailed structural comparison reveals that the conformation of the β3/β4 and β6/β7 loops in SrtE1 are distinct (Fig 3B). Based on our previously determined structures of class A and B enzymes bound to their substrates, these loops in SrtE1 are presumably part of the binding site for the LAXTG sorting signal [37–39,44]. In Fig 3B, the structures of these loops in SrtE1 are compared to the corresponding loops of the class A and B enzymes from *S*. *aureus*, which we have structurally characterized bound to their sorting signals [37,38]. It is readily apparent that the β6/β7 (blue) and β3/β4 (green) loops in SrtE1 are positioned closer to one another, resulting in a more confined binding site for the sorting signal (Fig 3B). Notably, the β3/β4 loop in SrtE1 is slightly longer than in other sortase enzymes, and as described below, contains a conserved tyrosine residue that may enable class E enzymes to recognize LAXTG sorting signals. Furthermore, the β6/β7 loop in SrtE1 contains a 21 amino acid insertion relative to class A enzymes that immediately follows a 3_10_ helix positioned adjacent to the active site cysteine (yellow) (Fig 3B). This long insertion is similar in length to that observed in class B sortases, but is distinctly devoid of secondary structure, whereas class B sortases contain an additional alpha helix.

### A distinct subsite on SrtE1 enables it to accommodate an alanine residue at position P3 in the sorting signal

**Fig 4:**
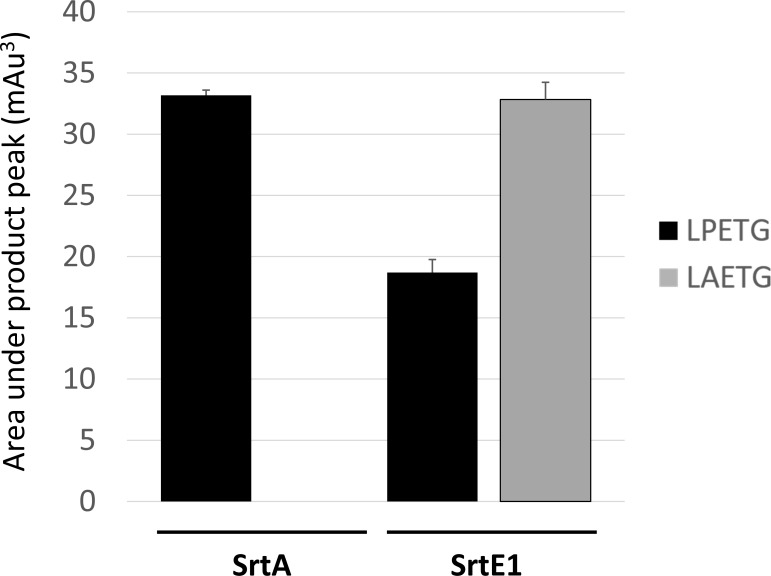
SrtE1 exhibits specificity for alanine at site P3 in the sorting signal. Hydrolysis activity of SrtE1 or SaSrtA towards LPETG or LAETG peptide substrates was determined with an established *in vitro* HPLC assay [48]. The enzyme cleaves the peptide at the threonine-glycine scissile bond, producing N- and C-terminal peptide products. The extent of hydrolysis was measured by integrating the area of the N-terminal peptide product peak in the HPLC chromatogram. Error bars indicate the standard deviation of integrated HPLC peak area obtained from duplicate hydrolysis reactions.

Previously, we demonstrated that SrtE1 attaches proteins to the cell wall that contain a novel LAXTG sorting signal [11]. All sortases characterized to date recognize protein sorting signals that contain a conserved PXT motif at their core (i.e. LPXTG for class A enzymes). Following convention, residues in the sorting signal are numbered based on their positioning relative to the scissile Thr-Gly peptide bond, where residues in the sequence L-P-*X*-T-G sorting signal are referred to as P4-P3-P2-P1-P1’, respectively (sites on the enzyme that recognize these residues are referred to as S4-S3-S2-S1-S1’, respectively). The P3 site within the sorting signal is important for substrate recognition by other sortase enzymes, as biochemical studies have shown that mutation of this residue to alanine disrupts the ability of class A and B sortases to process their substrates [38,46]. Interestingly, the sorting signals recognized by SrtE1 are highly unusual as they contain an alanine at position P3, which contrasts ~90% of all known sorting signals that contain a proline. The distinct substrate specificity of SrtE1 appears to be a hallmark of class E enzymes, as comparative genome analyses predict that they also attach proteins to the cell that contain sorting signals with an alanine residue at position P3 [36]. To explore SrtE1’s selectivity for alanine, its ability to cleave peptides containing the sequence LPETG or LAETG was determined. Each peptide was incubated with the enzyme separately, and the reaction products were separated by HPLC [42,47]. Interestingly, SrtE1 is capable of cleaving both peptides at the T-G peptide bond, as verified by MALDI (data not shown), but it exhibits a 2-fold preference for alanine at site P3 as compared to proline (Fig 4). This promiscuity is unique, as similar studies using the class A SaSrtA enzyme revealed that it could only hydrolyze the peptide containing a proline at position P3, whereas the alanine containing peptide was enzymatically inert (Fig 4) [46]. The selectivity of SaSrtA for proline is not surprising, as a structure of the enzyme bound to its sorting signal reveals that this residue enables the peptide to adopt an ‘L’-shaped conformation that is complementary to the enzyme’s active site [37,39].

**Fig 5:**
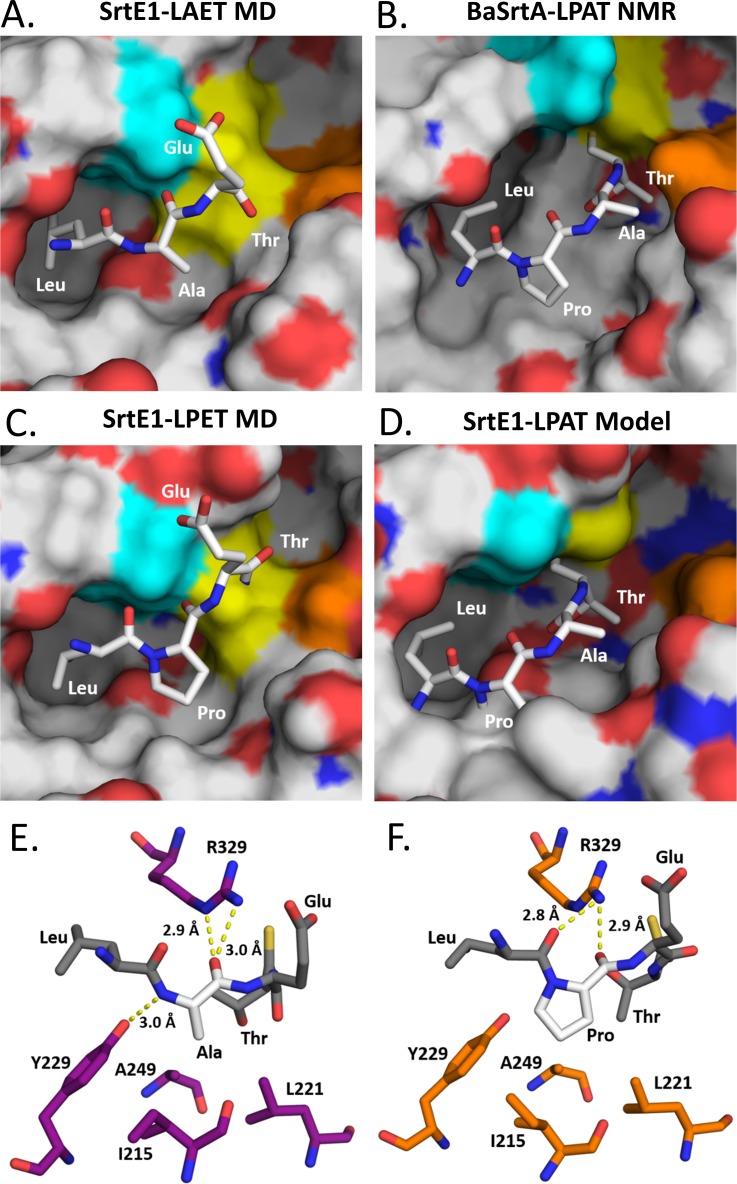
Energy minimized models of SrtE1-substrate complexes provide insight into the mechanism of recognition of the LAXTG sorting signal. A) Model of SrtE1 binding the LAET motif. The LAET peptide (*gray sticks*) was docked to the SrtE1 active site (*electrostatic surface*) using GLIDE and energy minimized through molecular dynamics simulations with NAMD2 [49–51]. The exposed surfaces of the catalytic residues Arg329 (*cyan*), Cys320 (*yellow*), and His251 (*orange*) residues are shown. B) NMR solution structure of SrtA from *B*. *anthracis* bound to LPAT substrate. The LPAT substrate mimic (*white sticks*) is positioned within the BaSrtA active site groove (*electrostatic surface*), defined by the exposed surfaces of the catalytic residues Arg196 (*cyan*), Cys187 (*yellow*), and His126 (*orange*) residues. C) Model of SrtE1 binding the LPET motif. The LPET peptide (*gray sticks*) was docked to the SrtE1 active site (*electrostatic surface*) using GLIDE and energy minimized through molecular dynamics simulations with NAMD2. The exposed surfaces of the catalytic residues Arg329 (*cyan*), Cys320 (*yellow*), and His251 (*orange*) residues are shown. D) Model of SrtE1 binding the canonical SrtA substrate motif. The catalytic cores of the SrtE1 crystal structure and solution structure of SrtA-LPAT* from *B*. *anthracis* (BaSrtA) (PDB ID: 2RUI) were structurally aligned in Pymol. The LPAT substrate mimic from BaSrtA (*white sticks*) clashes with the SrtE1 active site groove (*electrostatic surface*), defined by the exposed surfaces of the catalytic residues Arg329 (*cyan*), Cys320 (*yellow*), and His251 (*orange*) residues. E) Hydrogen bond interactions between SrtE1 active site residues and LAET substrate motif. SrtE1 residues within 4 angstroms of the LAET peptide are shown (*magenta sticks*). Energy minimized LAET peptide (*gray sticks*) containing the unique alanine residue (*white sticks*) is indicated. Hydrogen bonds (*yellow dashed lines*) between R329, Y229, and the energy minimized LAET peptide backbone are shown. F) Hydrogen bond interactions between SrtE1 active site residues and LPET substrate motif. SrtE1 residues are shown (*orange sticks*). Energy minimized LPET peptide (*gray sticks*) containing a proline residue (*white sticks*) is indicated. Hydrogen bonds (*yellow dashed lines*) between R329 and the energy minimized LPET peptide backbone are shown.

To gain insight into SrtE1’s unique ability to preferentially recognize sorting signals containing alanine at position P3, we computationally modeled how it binds to its LAETG and LPETG substrates. This work leveraged our experimentally determined structure of the class A sortase from *B*. *anthracis* (BaSrtA) bound to an analog of its sorting signal that contains a proline at position P3 (the BaSrtA-LPAT* complex) [39]. We modeled the SrtE1-substrate thioacyl complexes formed by these peptides, as it is a long-lived reaction intermediate that forms immediately after cleavage of the T-G peptide bond; in the reaction intermediate, the threonine carbonyl atom in the sorting signal was joined to the active site cysteine residue in SrtE1 via a thioacyl bond. Details of the modeling procedure are presented in the Methods section. Briefly, modeling involved positioning the peptide in the active site of SrtE1 using ligand docking and peptide coordinates derived from the structure of the BaSrtA-LPAT* complex, *in silico* construction of the thioacyl linkage, and solvated molecular dynamics (MD) and energy minimization calculations. Fig 5 shows the models of the SrtE1-LAET (Fig 5A) and SrtE1-LPET (Fig 5C) thioacyl intermediates. In both models, the bound sorting peptide adopts an ‘L’ shape as a result of a kink at the P3 residue. The non-polar side chains of either the alanine or proline residue at position P3 packs against Ala249 in the underlying β-sheet, as well as Ile215 and Leu221 within the β2-H2 loop. As observed in BaSrtA-LPAT (Fig 5B), the proline residue in the peptide of SrtE1-LPAT adopts a trans conformation producing an inherent kink that redirects the chain (Fig 5D), whereas in the LAET peptide, the kink occurs when the alanine residue adopts semi-favorable -57.9° phi and 173.6° psi torsional angles (Fig 5A). In both peptides, the kink causes the leucine P4 side chain to project into a hydrophobic S4 subsite on SrtE1 that is formed by residues located on the β7 (T318) and β8 strands (I331), as well as the N-terminal end of the β6/β7 loop (T291, P293, S294, N295, V296, V298, and L299). Specifically, the P4 leucyl side chain packs against the non-polar side chains of Val296, Val298 and Leu299 in the 3_10_ helix H4, as well as the side chain of Thr291 and α-protons of Pro293, Ser294 and Asp295 in the β6/β7 loop. Not surprisingly, the overall conformation of the bound peptide and the positioning of the P3 and P4 residues in the SrtE1-peptide models are generally similar to the sorting signal positioning observed in the experimentally determined structures of the BaSrtA, SaSrtA and SaSrtB enzyme-substrate complexes [37–39]. However, in the case of the SaSrtB, a hydrophilic threonine residue (T177) is present within the β6/β7 loop to coordinate the polar asparagine at position P4 within its NPQTN sorting signal, whereas in the SrtE1-peptide models, the S4 subsite is non-polar so as to interact with the leucine side chain in the LAETG and LPETG sorting signals (Fig 5A and 5C).

The S3 subsite of SrtE1 contains a conserved tyrosine residue that may enable it to preferentially recognize alanine instead of proline at site P3 in the sorting signal. The tyrosine residue, Tyr229, is located in the β3/β4 loop and, along with the side chain of Ile215 in the β2/H2 loop, forms a unique SrtE1-specific ridge in the S3 subsite. In the energy minimized models of the SrtE1-LAET and SrtE1-LPET complexes, the S3 subsite can readily accommodate the methyl and pyrrolidine ring side chains of their respective sorting signals (Fig 5E and 5F, respectively). In particular, the S3 subsite forms hydrophobic contacts to these side chains via its Ile215, Leu221 and Ala249 residues, while the active site arginine residue (R329) donates a hydrogen to the acceptor carbonyl oxygen of the P3 residue via its guanidino group (Fig 5E and 5F), forming a hydrogen bond. These interactions have also been observed in the experimentally determined structures of sortases bound to their substrates [37–39]. Intriguingly, the tyrosine residue in the S3 subsite appears to preferentially stabilize the alanine-containing sorting signal. As shown in the energy minimized model of the SrtE1-LAET complex (Fig 5E), the backbone amide nitrogen of the P3 residue is positioned to donate a hydrogen to the acceptor Tyr229 hydroxyl, forming a hydrogen bond. This hydrogen bond may preferentially stabilize binding to the alanine containing peptide, as a proline residue at this site would contain a nitrogen atom that would be unable to act as a hydrogen bond donor. Notably, the binding mode of the peptide in the SrtE1-LAET model is similar to that of the AQA tripeptide in the crystal structure of SrtE1, suggesting that it is a biologically accessible conformation (Fig 2C). Unfavorable enzyme-substrate steric interactions involving the tyrosine residue may also further discourage binding of sorting signals that contain a proline residue at P3. This is demonstrated in Fig 5B and 5D, where we compared the experimentally determined structure of the BaSrtA-LPAT* complex and a model of SrtE1-LPAT complex in which the peptide had been simply placed into the enzyme active site in an identical manner as in BaSrtA without any energy refinement (SrtE1-LPAT model). Unlike the LPAT peptide bound to BaSrtA, which is complementary to the enzyme’s active site, the P3 proline residue in the non-energy minimized SrtE1-LPAT model sterically clashes with ridge atoms within the S3 subsite of SrtE1 (Fig 5D). These unfavorable contacts can only be alleviated by energy minimization of the atomic coordinates (Fig 5C). Interestingly, even though energy minimization enables the proline residue to properly fit into the S3 subsite, the P4 leucine side chain is not fully ensconced within the hydrophobic S4 subsite. Together, the modeling data suggests that steric hindrance and hydrogen bonding imparted by the tyrosine residue in the S3 subsite may cause SrtE1 to preferentially recognize sorting signals that contain an alanine residue at position P3.

Several indirect lines of evidence support the notion that class E enzymes like SrtE1 use a conserved tyrosine residue to recognize sorting signals that contain an alanine at position P3. First and foremost, an amino acid sequence alignment reveals that the tyrosine residue at this position is highly conserved in class E enzymes that are predicted to recognize LAXTG sorting signals, while it is frequently absent in other types of sortases [3,35,36] (S3 Fig). Second, several biochemical studies of A, B and D sortases that do not contain the analogous tyrosine residue in their S3 subsites have revealed that they are unable to process signals containing alanine at site P3 [38,46,52]. Interestingly, these studies have shown that even conservative mutation of the tyrosine residue may disrupt alanine recognition. In particular, recent studies of the *Clostridium perfringens* SrtD enzyme (CpSrtD) that contains a phenylalanine (F92) instead of tyrosine residue have shown that it preferentially cleaves the sequence LPQTGS motif, but does not process an LAETG sorting signal [35,52]. Notably, CpSrtD was originally classified as a class E enzyme, but was later re-classified using hidden Markov models to be a class D enzyme [35,36]. This is consistent with our assertion that tyrosine plays an important role in signal recognition, as CpSrtD also lacks the class E specific tyrosine residue within its S3 subsite. Third, the results of directed evolution studies of the class A SaSrtA enzyme are compatible with the proposed substrate specificity determinant role of the conserved tyrosine residue in SrtE1. Specifically, Dorr et al. observed a marked shift in the specificity of evolved SaSrtA enzymes to preferentially cleave LAETG over LPETG substrates once a mutation was acquired within the β3/β4 loop at an analogous site to Y229 in SrtE1 (SaSrtA A104H mutation) [53]. These observations suggest that selectivity for an alanine at the S3 subsite could be mediated by the presence of a residue with a bulky, aromatic side chain and a hydrogen bond acceptor group within the β3/β4 loop; these features would partially exclude proline from the active site and stabilize the peptide backbone amide or carbonyl groups of substrates containing a residue with a small, nonpolar side chain. Unfortunately, our attempts to experimentally probe the dependence of substrate specificity on the tyrosine residue were unsuccessful, as SrtE1 proteins containing single amino acid mutations that change its Tyr229 residue to either phenylalanine or alanine are unstable (data not shown).

In conclusion, work presented in this paper has revealed unique class E sortase features within the catalytic domain of the SrtE1 enzyme from *S*. *coelicolor* and highlighted the functional importance of N-terminal cytoplasmic residues. Our biochemical studies indicate that SrtE1 can recognize sorting signals that contain either alanine or proline at position P3. Based on models of its reaction intermediates, we propose that SrtE1 and other class E sortases recognize unique alanine containing sorting signals by employing a conserved tyrosine residue within their β3/β4 loops. The tyrosine presumably biases recognition for alanine through a combination of steric effects and hydrogen bonding. However, it is important to stress that residues in addition to tyrosine may also be needed to confer enzyme specificity for alanine at site P3, as directed evolution studies of the SaSrtA enzyme have shown that a set of 11 mutations are required to change its specificity from LPXTG to LAXTG [53]. The majority of these mutations were in the peptide binding pocket and included an A104H alteration in the S3 subsite at a position that is analogous Tyr229 in SrtE1 [53]. Notably, guided by these studies, we attempted to bias the specificity of SaSrtA for alanine at site P3 by introducing a single A104Y mutation. However, this single amino acid mutant was unable to recognize both LPXTG and LAXTG substrates, suggesting that more than one mutation in the enzyme’s binding pocket is required to change its substrate specificity (data not shown). Combined, the data suggest that binding of the sorting signal is a complex process, whose specificity is dictated by multiple, interdependent interactions with amino acids in the enzyme. Elucidating the determinants of sortase substrate specificity will require additional atomic structures of sortases bound to their substrates and the application of more sophisticated computational modeling approaches. The results of this work will facilitate prediction of sortase function among a wide range of microbes, rational design of substrate-based inhibitors that could function as antibiotics, and engineering of sortases with altered specificities that could have useful biotechnological applications.

## Materials and Methods

### Cloning, expression, and protein purification

The extracellular domain of SrtE1 from *S*. *coelicolor* (SrtE1^ΔN^, residues T162-S352) was expressed from a pET-15b plasmid in *Escherichia coli* BL21(DE3) cells. Standard methods were employed, with cultures grown in the presence of ampicillin at 37°C until an OD_600_ of 0.6–0.8 was reached. Protein expression was then initiated by adding 100 μM isopropyl- β-D-1-thiogalactopyranoside (IPTG) followed by overnight protein expression at 25°C. A two liter cell culture was harvested by centrifugation and re-suspended in 40 mL of lysis buffer (50 mM Tris, pH 7.5; 300 mM NaCl) that contained 1 mg/mL lysozyme (Sigma-Aldrich), 400 μl of protease inhibitor cocktail (Calbiochem) and 2 mM phenylmethanesulfonylfluoride (PMSF). The cells were then incubated on ice with stirring for ~30 min and further lysed by sonication. Cell lysates were fractionated by centrifugation and the soluble portion applied to a gravity column containing 10 mL of suspended His-Pure Co^2+^ resin (Life Technologies) pre-equilibrated with lysis buffer. The resin was sequentially washed with 20 mL aliquots of lysis buffer that contained 0, 10, and 25 mM imidazole. His-tagged SrtE1 was then eluted using 500 mM imidazole, and the fractions pooled, concentrated, and buffer exchanged into 50 mM Tris, pH 7.5; 150 mM NaCl using an Amicon Ultra-15 centrifugal filter (Millipore). To remove His_6_-tag from the protein, one unit of thrombin protease (GE Healthcare) was added for every 100 μg of SrtE1, and the solution was incubated at 4°C overnight. Thrombin was then separated from SrtE1 using a HiTrap-Benzamidine column (GE Healthcare). Specifically, the SrtE1-thrombin mixture was loaded onto the column using Buffer A (50 mM Tris, pH 7.5; 150 mM NaCl), followed by washing with Buffer A and subsequent addition of Buffer B (50 mM Tris, pH 7.5; 1 M NaCl) to recover absorbed SrtE1. SrtE1 lacking the His_6_-tag was further purified by gel filtration chromatography using a Sephacryl size-exclusion column (GE Healthcare Life Sciences) equilibrated in 50 mM Tris, pH 7.5 and 150 mM NaCl. Purified SrtE1 was then pooled, concentrated to 7 mg/mL, and stored at 4°C.

Three single amino acid mutants of SrtE1^ΔN^ were constructed, using the extracytoplasmic sequence as a starting point. Two point mutant variants targeting Y229 were generated using site-directed mutagenesis (mutated codon is underlined). Y229A mutation was generated by PCR amplification using forward (CGGGGCATGGTCGGGCACGCCGCGGAGGACGGGCTGAAG) and reverse (CTTCAGCCCGTCCTCCGCGGCGTGCCCGACCATGCCCCG) primers. Y229F mutation was generated by PCR amplification using forward (CGGGGCATGGTCGGGCACTTCGCGGAGGACGGGCTGAAG) and reverse (CTTCAGCCCGTCCTCCGCGAAGTGCCCGACCATGCCCCG) primers. An N-terminally truncated version was also generated, such that the first 34 extracytoplasmic amino acids were removed from the overexpression construct. This construct essentially recapitulated the portion of SrtE1 that was crystallized, and was generated by PCR amplification using the primers SrtE1 short NdeI (GGTCGCATATGGGCATCGGCTTCCTGCACG; NdeI site is underlined), and 3850 BamHI (GGGTGCGGATCCTTAACTGACGAGCGCATCC; BamHI site is underlined), using the original overexpression construct as template. The resulting PCR product was digested with NdeI and BamHI, cloned into pET15b digested with the same enzymes, and sequenced to confirm insert integrity. Protein overexpression was achieved as described above.

### Crystallization, Data Collection, and Structural Determination

Recombinant SrtE1^ΔN^ at a concentration of 7 mg/mL in 50 mM Tris, pH 7.5; 150 mM NaCl was used for crystal screening. Screening used the Structure Screen broad matrix suite (Hampton Research) at room temperature in a sitting-drop vapor diffusion format (200 nl drop size). Protein crystals grew over the course of 3 to 4 weeks or longer in the presence of 50 mM HEPES, pH 7.5, 1.4 M sodium citrate and reached dimensions of ~ 0.10 mm x 0.05 mm. For X-ray data collection, SrtE1 crystals were cryoprotected using reservoir solution containing 30% glycerol. Diffraction data sets were collected at the Advanced Photon Source (APS) beamline 24-1D-C equipped with a Pilatus-6M detector. All data were collected at 100 K. Data were collected at the detector distance of 600 mm, with 0.5° oscillations, and at a 0.9791 Å wavelength. The crystals diffracted X-rays to 1.8 Å resolution. The XDS/XSCALE package was used to index, integrate and scale data in C222_1_ space group [54]. The asymmetric unit of the crystal contained a single protein molecule, yielding a Matthews coefficient of 2.57 Å/Da and a 52.23% solvent content in the crystal. The PHASER program was used for molecular replacement, employing the coordinates of the SrtC1 enzyme from group B streptococcus as a search model (PDB ID: 4G1J); loops within the search model that had high B-factors were deleted [55]. Molecular replacement yielded a single solution, which was refined in iterative runs using PHENIX software [56]. Modeling of the additional active site density was confirmed using 2F_o_-F_c_ omit maps generated using PHENIX [56]. Complete refinement and structure statistics are reported in Table 1. The high value for the ligand B-factor indicated incomplete occupancy of a glycerol molecule within the crystal lattice. Coordinates and structure factors have been deposited in the Protein Data Bank with accession code 5CUW.

### Computational Modeling and Molecular Dynamics Simulations

Models of the thioacyl intermediate containing SrtE1 bound to either LPET or LAET peptides were generated. The procedures used to construct these models have been described previously [38,39]. Briefly, the coordinates of each peptide were derived from the coordinates of the LPAT peptide in the NMR structure of the BaSrtA-LPAT complex [39]. The LPET or LAET coordinates were created by *in silico* mutation of the LPAT coordinates using Pymol. The LPET or LAET peptides were then docked to the crystal structure of SrtE1 using the Schrödinger Small-Molecule Drug Discovery Suite 2015–2 (Schrödinger LLC, New York, NY, USA). Prior to docking, the LPET and LAET ligands were constructed and energy minimized with LigPrep and the AQA tripeptide was removed from the crystal structure of the enzyme. The receptor (SrtE1) was also processed with the Protein Preparation Wizard to add missing side chain atoms and hydrogens, and to perform a restrained, partial energy minimization of the coordinates [57]. Docking grid was generated using Glide [49,50], and had dimensions of 22 x 22 x 22 Å centered around the active site. Docking was performed with Glide in SP Peptide docking mode using default settings [58]. A single model with the lowest docking energy for LPET and LAET peptides separately and the carboxyl group of Thr in the peptide within 3 Å of Cys320 were further refined using MD simulations.

The procedures used for the MD simulations have been described previously [38,39]. Briefly, the carboxyl oxygen on the Thr residue within the docked peptide was modified to enable thioacyl bond formation to the active site cysteine (C320). Parameters for the thioacyl linkage had been generated from our previous study [38,39]. Using tLeap, the models were first solvated in a triclinic box of TIP3P water molecules with sufficient sodium and chloride ions to create a neutral simulation box of approximately 150 mM NaCl [59]. Models were then energy-minimized and equilibrated in NAMD 2.6 using the AMBER14SB force field by slowly removing restraints from the initial atom positions over 1 ns (2-fs step size) [38,51]. For each enzyme-peptide model, two sequential 10-ns MD simulations were performed.

### In vivo functional assay for SrtE1 activity

To probe the functional significance of the cytoplasmic N-terminal extension of SrtE1, we created three different mutant variants (Δ14–113, Δ82–90 and Δ15–79), and tested their ability to complement the developmental defects of a *srtE1-srtE2* mutant. Two of the mutant constructs were synthesized (Δ14–113 and Δ15–79) (Genscript), and cloned into pUC57. These constructs were then excised using HindIII and KpnI, and were cloned into pMS82 digested with the same enzymes [60]. The resulting constructs were confirmed by sequencing, prior to being conjugated into the *S*. *coelicolor* Δ*srtE1/E2* mutant strain, where they were integrated into the chromosome [61]. The other mutant (Δ82–90) was generated by site-directed mutagenesis, using pMC134 (pMS82 containing *srtE1*) as template, together with the primers 3850 del4-F (GGGGGCGGTCCGGACGGCGGCGGT**CGGCGTCACGGGCGCCGTGGGGCG**) and 3850 del4-R (CGCCCCACGGCGCCCGTGACGCCG**ACCGCCGCCGTCCGGACCGCCCCC**), where each primer encompasses sequences flanking the deleted region (underlined and bolded sequences delineate the two sides of the deleted region) [61]. Following PCR amplification, the resulting product was treated with DpnI, and introduced into *E*. *coli* XL1-Blue cells by electroporation. The sequence of the deletion construct was confirmed by sequencing, after which the plasmid was introduced by conjugation into the double sortase mutant strain. The ability of the different mutant derivatives was assessed using phenotypic analyses of the strains grown on sporulation (MS) medium, comparing each strain with the *srtE1/E2* mutant strain containing either an empty plasmid vector (pMS82) or one bearing a wild type version of *srtE1* (pMC134) [62].

### In vitro hydrolysis assay for substrate recognition and cleavage

The *in vitro* hydrolysis reactions were performed as described by Kruger et al. [48]. Ten micromolar sortase enzyme (wild-type or mutant) was incubated with 100 mM peptide substrate in 100 μl of assay buffer (50 mM Tris-HCl, 150 mM NaCl, 5 mM CaCl_2_, 1 mM DTT) at 25°C for 72 h. The reactions were quenched by adding 50 μl of 1 M HCl and injected onto a Waters XSelect HSS C18 reversed phase HPLC column. Peptides were eluted by applying a gradient from 0 to 40% acetonitrile (in 0.1% trifluoroacetic acid) over 40 min at a flow rate of 1 ml/min. Elution of the peptides was monitored by absorbance at 215 nm. Peak fractions were collected, and their identities were confirmed by MALDI-TOF mass spectrometry. The amount of product produced was determined by integrating the area of the product peak in the HPLC trace.

## Supporting Information

S1 Fig Alignment of the N-terminal extension of SrtE1 orthologues in diverse *Streptomyces* species. Sequence alignment was generated using the ClustalOmega server [43]. The bacterial species and accession numbers of the amino acid sequences used for the alignment are as follows: *Streptomyces clavuligerus* (EDY51820), *Streptomyces coelicolor* (WP_011029270), *Streptomyces viridochromogenes* (EFL33429), *Streptomyces sviceus* (WP_007383307), *Streptomyces ghanaensis* (WP_004985874), *Streptomyces griseoflavus* (WP_040906697), *Streptomyces scabiei* (WP_013002225), *Streptomyces albus* (WP_015507549). Conserved residues are indicated in *red*, and related amino acids are indicated in *blue*. The conserved acidic and basic regions are boxed in *red* and *blue*, respectively. The conserved transmembrane helix is boxed in *green*.
(PDF)

S2 Fig ^1^H-^15^N HSQC spectrum of the SrtE1 extracellular domain construct. The ^1^H-^15^N HSQC spectrum yielded reasonably well-resolved cross peaks, indicating that the SrtE1 protein was folded. However, there were substantially fewer peaks than anticipated for the molecular weight of the SrtE1^ΔN^ construct (20.8 kDa). In particular, ~29 peaks were absent in the NMR spectra; only ~148 resolvable cross peaks from backbone amides were observed, whereas 177 cross peaks are expected (194 total residues– 16 proline residues–the N-terminal residue). The reduced number of signals in the ^1^H-^15^N HSQC spectrum is compatible with the N-terminal linker experiencing motions that are intermediate on the chemical exchange time scale (μs to ms), causing their signals to be broadened. Such motions are also compatible with our inability to visualize residues from the N-terminal linker (with the exception of the AQA tripeptide) within the electron density map. Combined, the NMR and crystallography data suggest that the AQA tripeptide is housed in a structurally disordered segment of the isolated enzyme. ^15^N-labeled SrtE1^ΔN^ for NMR studies was concentrated to 350 μM in NMR buffer (50 mM NaPO4, pH 6.8; 150 mM NaCl, 7% D_2_O). HSQC spectra were acquired with 32 scans at 298 K on Bruker 600 MHz spectrometers equipped with a triple-resonance cryogenic probe.
(PDF)

S3 Fig Alignment of phylogenetically determined class E enzymes with SrtD from *Clostridium perfringens*. Sequence alignment was generated using the ClustalOmega server [43]. The bacterial species and accession numbers of the amino acid sequences used for the alignment are as follows: *Streptomyces coelicolor* (NP_628038 and NP_628037), *Bifidobacterium longum* (NP_695779), *Corynebacterium diptheriae* (NP_940575), *Corynebacterium efficiens* (NP_739396), *Corynebacterium glutamicum* (NP_602126), *Streptomyces avermitilis* (NP_825514; NP_826383; NP_825510), *Streptomyces griseus* (YP_001825232; YP_001825235; YP_001826193; YP_001825236), *Thermobifida fusca* (YP_290439), *Tropheryma whipplei* (NP_787692), *Clostridium perfringens* (WP_003467492), *Clostridium tetani* (WP_011099430). Conserved residues are indicated in *red*, and related amino acids are indicated in *blue*. The conserved tyrosine residue within the B3/B4 loop of class E sortases is boxed in *black*.
(PDF)
