# Supplementary material for: Crystal Structure of the Streptomyces coelicolor Sortase E1 Transpeptidase Provides Insight into the Binding Mode of the Novel Class E Sorting Signal
Source: PLoS One. 2016 Dec 9;11(12):e0167763. doi: 10.1371/journal.pone.0167763 (PMC5148588; doi:10.1371/journal.pone.0167763)
Supplement: S1 Fig — Sequence alignment was generated using the ClustalOmega server [43]. The bacterial species and accession numbers of the amino acid sequences used for the alignment are as follows: Streptomyces clavuligerus (EDY51820), Streptomyces coelicolor (WP_011029270), Streptomyces viridochromogenes (EFL33429), Streptomyces sviceus (WP_007383307), Streptomyces ghanaensis (WP_004985874), Streptomyces griseoflavus (WP_040906697), Streptomyces scabiei (WP_013002225), Streptomyces albus (WP_015507549). Conserved residues are indicated in red, and related amino acids are indicated in blue. The conserved acidic and basic regions are boxed in red and blue, respectively. The conserved transmembrane helix is boxed in green. (PDF) [file pone.0167763.s001.pdf]

```
S.clavuligerus      1 ------------------------------------------------------------
S.coelicolor        1 ------------------------------------------------------------
S.viridochromog     1 -----------------------------MPGAFEDWPAGGEYGASPQPEAQPQPGSYAP
S.sviceus           1 -------------------------MTALRPEREDLYGDASYESYGGDPYAGHSFGGASY
S.ghanaensis        1 ---------------------------------------------MTALRPERESGAGYG
S.griseoflavus      1 -------------------MAGGVRRRGRGDEVGGARPGGGQVLGVTALRPERESGA---
S.scabiei           1 MTALRPERESAPYG-GEAAYGGAEAFEAETTFEPGVPFRDPAEPQQWAAPQASPYAQGDW
S.albus             1 MTALRPERESAAGGPVDPLTDPLPDVLAGGHSSPWFRAEQPPQLVQGHDLSAPQPPLADP

S.clavuligerus      1 -----------------------MHPPGVPPAPAPEQWG----TPRPPGQPEPPGPYEPP
S.coelicolor        1 -------MTALRPERDSGTAGDQGSSYGQPYGDSGAFGGGRYEESAAGEENRPPLLDDET
S.viridochromog    32 RPESYAPQSESYAPQPGSYVSQ----PEPYSASYEPLPDGSLPDESLPGESYLPPVDEET
S.sviceus          36 AEEPYAESPYGEEPYAESSYAEGQYTEQPPGGVSEGRASDGGASDRQASAPYVPPADDET
S.ghanaensis       16 ESFTDHPGYGVGGEQGTPYGQQPYGAPGTFGDGRYDGAPRDPYGGASDDTAYLPPVDEET
S.griseoflavus     39 ------DAYGGAGGQGTSYGGQPHGAPGAYGDGWYDTTAPGPHGGSSDTTAYLPPVDEET
S.scabiei          60 HGQQPYAEPAFGSYETYGQRPQAPYEGRPPASYDLYAAAGVQAGLPADTAQEHPPIDEET
S.albus            61 YAQLP-ADAYAAPPQDDAYAGHSPYEGGPGWTASPETYPAPEAAEEPYSPVPEAPRDDET

S.clavuligerus     34 ETYEPYEPYEP------YEHAPTGPTA--PTGPGRAERRRAARSPGHRAR----RRPPKP
S.coelicolor       54 VALRIPEPPAP------RTAAGTGPIGGGPDGGGRAARRKAAKRRHGRRG----APRDQA
S.viridochromog    88 VALRIPDPPPSGSDAVSGSPASSATRRDDPSQGGRAARRKAAKRRHGRHG----GAPDTG
S.sviceus          96 VALRIPDPPPP---SISASTASSVTSATGSPQGGRAARRKAAKGRHGRHG----GGAAP-
S.ghanaensis       76 VALRIPDPPDP--------PERPVREEPTGAPGGRAARRKAAKRRQGRRG----SPRGAG
S.griseoflavus     93 VALRIPEPPER--------TAR--EEPAPAAPGGRAARRKAAKKRQGRRG----GSHKAA
S.scabiei         120 VALRVEEARRAA----ARAESIPPSAVTAG----RAARRKAARRHGRHGG---SPAAHAA
S.albus           120 MALRAADPARTD----EERDETPPATISAGAEGGRAARRKAAKRNARPGGRRGTGRRRAG

S.clavuligerus     82 SPQATAPS----------RPLTRTEARRAARAARDSPGVVVSRALGEVFITLGVVMLLFV
S.coelicolor      104 PEEEAEQAP--------KAPLSRVEARRQARARKPGAAVVASRAIGEIFITTGVLMLLFV
S.viridochromog   144 PAPEGAREE-------RRAPLSRVEARRQARARKPSPAVVASRAIGEVFITTGVLMLLFV
S.sviceus         148 EAQSDQESP-------DGRPLSRVEARRQAKARKPGAAVVASRAIGEVFITTGVLMLLFV
S.ghanaensis      124 EPEAASRAP----EGPSGAPLSRVEARRRARARRASPAVVASRAIGEVFITTGVLMLLFV
S.griseoflavus    139 EAPRDSRGPDGGSEGASRAPLSRVEARRRARASKASPAVLASRAIGEVFITCGVLMLLFV
S.scabiei         169 SRAAQGPGQPLGAAPSASAPLSRIEARRAARAQKPGVGVIASRVIGEVFITTGVVMLLFV
S.albus           176 APAPEASGTPGGGDEPPERPRTRVEARRAARARKPSAATVASRAVGELFITIGVLMLLFV

S.clavuligerus    132 TYQLWWTNVRADQQAGREKERIQRSWAAG-RAPGAFRPGEGFAIMYIPKLDVVVPVAASI
S.coelicolor      156 TYQLWWTNVRAHAQANQAASNLQDDWANGKRSPGSFEPGQGFALLHIPKLDVVVPIAEGI
S.viridochromog   197 TYQLWWTNVRAHAQAGSEASSLQNDWASGKRNPGAFEPGQGFAI----------------
S.sviceus         201 SYQLWWTNVRAHAQADKEASSLQNDWASGKGAPGTFEPGQ--------------------
S.ghanaensis      180 TYQLWWTNVRAQAQAGKELSDLQSDWANGKRNPGVFEPGQGFALLHIPKLDVVVPIAEGI
S.griseoflavus    199 TYQLWWTNVRAHAQAGKEVSDLQSDWANGAGKPGAFEPGQGF------------------
S.scabiei         229 TYQLWWSNIRAH------------------------------------------------
S.albus           236 TYQLW-------------------------------------------------------
```

Fig S1.
