# Supplementary figures and images for: Crystal Structure of the Streptomyces coelicolor Sortase E1 Transpeptidase Provides Insight into the Binding Mode of the Novel Class E Sorting Signal
Source: PLoS One. 2016 Dec 9;11(12):e0167763. doi: 10.1371/journal.pone.0167763 (PMC5148588; doi:10.1371/journal.pone.0167763)

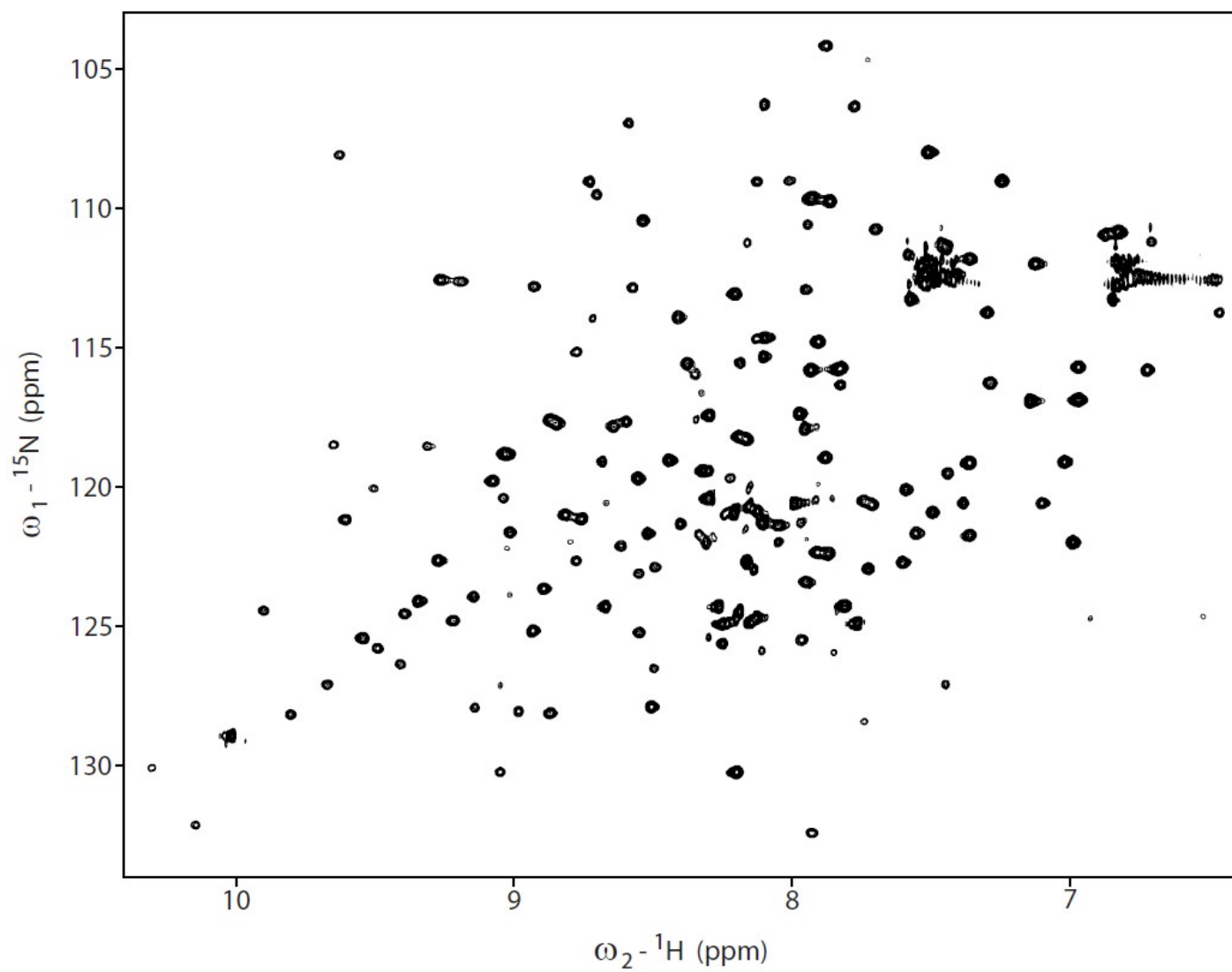

Fig S2.

Supplement: S2 Fig — The 1H-15N HSQC spectrum yielded reasonably well-resolved cross peaks, indicating that the SrtE1 protein was folded. However, there were substantially fewer peaks than anticipated for the molecular weight of the SrtE1ΔN construct (20.8 kDa). In particular, ~29 peaks were absent in the NMR spectra; only ~148 resolvable cross peaks from backbone amides were observed, whereas 177 cross peaks are expected (194 total residues– 16 proline residues–the N-terminal residue). The reduced number of signals in the 1H-15N HSQC spectrum is compatible with the N-terminal linker experiencing motions that are intermediate on the chemical exchange time scale (μs to ms), causing their signals to be broadened. Such motions are also compatible with our inability to visualize residues from the N-terminal linker (with the exception of the AQA tripeptide) within the electron density map. Combined, the NMR and crystallography data suggest that the AQA tripeptide is housed in a structurally disordered segment of the isolated enzyme. 15N-labeled SrtE1ΔN for NMR studies was concentrated to 350 μM in NMR buffer (50 mM NaPO4, pH 6.8; 150 mM NaCl, 7% D2O). HSQC spectra were acquired with 32 scans at 298 K on Bruker 600 MHz spectrometers equipped with a triple-resonance cryogenic probe. (PDF) [file pone.0167763.s002.pdf]
