# Supplementary material for: Crystal Structure of the Streptomyces coelicolor Sortase E1 Transpeptidase Provides Insight into the Binding Mode of the Novel Class E Sorting Signal
Source: PLoS One. 2016 Dec 9;11(12):e0167763. doi: 10.1371/journal.pone.0167763 (PMC5148588; doi:10.1371/journal.pone.0167763)
Supplement: S3 Fig — Sequence alignment was generated using the ClustalOmega server [43]. The bacterial species and accession numbers of the amino acid sequences used for the alignment are as follows: Streptomyces coelicolor (NP_628038 and NP_628037), Bifidobacterium longum (NP_695779), Corynebacterium diptheriae (NP_940575), Corynebacterium efficiens (NP_739396), Corynebacterium glutamicum (NP_602126), Streptomyces avermitilis (NP_825514; NP_826383; NP_825510), Streptomyces griseus (YP_001825232; YP_001825235; YP_001826193; YP_001825236), Thermobifida fusca (YP_290439), Tropheryma whipplei (NP_787692), Clostridium perfringens (WP_003467492), Clostridium tetani (WP_011099430). Conserved residues are indicated in red, and related amino acids are indicated in blue. The conserved tyrosine residue within the B3/B4 loop of class E sortases is boxed in black. (PDF) [file pone.0167763.s003.pdf]

```
Streptomyces_coelicolor_SrtE1       197 FALLHIPKLDVVV-PIAEGISSKKVLD---RGMVGHYAE----DGLKTAMPDAKAGNFGL
Streptomyces_coelicolor_SrtE2        96 IGFLHVPAMSEGD-ILVEKGTSMKILN---DGVAGYYTD----PVKATLPTSDEKGNFSL
Bifidobacterium_longum              140 VAQIYIPRFGSQWHRNIVEGTTLEQLN---RHGLGHYDT----T-----QMPGQVGNFAV
Corynbacterium_diptheriae            29 FARMYIPQFGSDFQFAIVEGTTDADLE---AGP-GHYND----T-----QLPGERGNFAV
Corynebacterium_efficiens           102 FARMYIPSFGSDFHFAVIEGTDEEELL---AGP-GRYTD----S-----QMPGEAGNFAV
Corynebacterium_glutamicum           86 FARMYVPAFGSDFNFAVIEGTDEEDLL---AGP-GRYVD----S-----QMPGEAGNFAV
Streptomyces_avermitilis_1           90 IGFLHVPAMNNGE-VLVRKGTSTQVLN---NGVAGYYTD----PVKAMLPMTGKNGNFTL
Streptomyces_avermitilis_2          124 YAVLTIPRLSLRV-PVAEGIGKASVLN---HGYVGHYPK----T-----AQPGRAGNFAL
Streptomyces_avermitilis_3           80 FAVMYIPRLGFTWNKPVLEGTGTEVLK---KGL-GHYAN----T-----ARLGQKGNFAV
Streptomyces_griseus_1               85 FATMHIPRFGADWEWPVLENTAVGTLK---KGL-GHYSA----T-----ARPGDTGNFAV
Streptomyces_griseus_2              319 FAIMHIPKLDVVA-PIAEGIDKEKVLD---RGMLGHYAE----GRLKTAMPSDKQGNFSV
Streptomyces_griseus_3              208 YAVLRIPRIGLTA-PVAEGTSKGGVLD---RGYVGHYAR----T-----AQAGQAGNFAL
Streptomyces_griseus_4               90 IGFLHVPAMKNGE-VLVKKGTDPETLN---NGIAGYYTD----PVESALPWD-DEGNFTL
Thermobifida_fusca                   99 NSRLYIPKTDQNW-VVVS-GVGPEDIK---YGP-GWYPESWTPEGMVPAARAGQPGNYAV
Tropheryma_whipplei                  97 IAVLFVPRFGNKYKRVIRETTDVTRVLNSKTAGVGHYPH----T-----ALPGTSGNFAV
Clostridium_perfringens_SrtD         43 IALIDIEKIGVHT--VIAEGSTLDVLE---N-NIGHFEN----T-----AMPGENGNFSI
Clostridium_perfringens             101 IGILNIPKINLEI--GIIEGVSYEDIK---Y-VVGHFPG----S-----PMPGEKGNFSI
Clostridium_tetani                   71 MAIIEIPSIGLKS--VIVEGTEMEKLR---Y-YIGHFKE----T-----ALPGQLGNFCI
```

Fig S3.
